# International evaluation of circumferential resection margins after rectal cancer resection: insights from the Swedish and Dutch audits

**DOI:** 10.1111/codi.14903

**Published:** 2019-11-27

**Authors:** R. Detering, D. Saraste, M. P. M. de Neree tot Babberich, J. W. T. Dekker, M. W. J. M. Wouters, A. A. W. van Geloven, W. A. Bemelman, P. J. Tanis, A. Martling, M. Westerterp, the Swedish ColoRectal Cancer Registry and the Dutch ColoRectal Audit, Arend Aalbers, Regina Beets‐Tan, Frank den Boer, Stephanie Breukink, Peter Paul Coene, Pascal Doornebosch, Hans Gelderblom, Tom Karsten, Michel Ledeboer, Eric Manusama, Corrie Marijnen, Iris Nagtegaal, Koen Peeters, Rob Tollenaar, Cock van de Velde, Anja Wagner, Erik van Westreenen

**Affiliations:** ^1^ Department of Surgery Amsterdam UMC University of Amsterdam Amsterdam The Netherlands; ^2^ Scientific bureau of the Dutch Institute of Clinical Auditing Leiden The Netherlands; ^3^ Department of Molecular Medicine and Surgery Karolinska Institutet, Stockholm Sweden; ^4^ Department of Gastroenterology and Hepatology Amsterdam UMC University of Amsterdam Amsterdam The Netherlands; ^5^ Department of Surgery Reinier de Graaf Hospital, Delft The Netherlands; ^6^ Department of Surgical Oncology Netherlands Cancer Institute–Antoni van Leeuwenhoek Hospital Amsterdam The Netherlands; ^7^ Department of Surgery Tergooi Hospital, Hilversum The Netherlands; ^8^ Department of Colorectal Surgery Haaglanden Medical Center The Hague The Netherlands

**Keywords:** Rectal neoplasms, hospitals, surgical margin, colorectal surgery, Sweden, Netherlands

## Abstract

**Aim:**

This study aimed to determine predictive factors for the circumferential resection margin (CRM) within two northern European countries with supposed similarity in providing rectal cancer care.

**Method:**

Data for all patients undergoing rectal resection for clinical tumour node metastasis (TNM) stage I–III rectal cancer were extracted from the Swedish ColoRectal Cancer Registry and the Dutch ColoRectal Audit (2011–2015). Separate analyses were performed for cT1–3 and cT4 stage. Predictive factors for the CRM were determined using univariable and multivariable logistic regression analyses.

**Results:**

A total of 6444 Swedish and 12 089 Dutch patients were analysed. Over time the number of hospitals treating rectal cancer decreased from 52 to 42 in Sweden, and 82 to 79 in the Netherlands. In the Swedish population, proportions of cT4 stage (17% *vs* 8%), multivisceral resection (14% *vs* 7%) and abdominoperineal excision (APR) (37% *vs* 31%) were higher. The overall proportion of patients with a positive CRM (CRM+) was 7.8% in Sweden and 5.4% in the Netherlands. In both populations with cT1–3 stage disease, common independent risk factors for CRM+ were cT3, APR and multivisceral resection. No common risk factors for CRM+ in cT4 stage disease were found. An independent impact of hospital volume on CRM+ could be demonstrated for the cT1–3 Dutch population.

**Conclusion:**

Within two northern European countries with implemented clinical auditing, rectal cancer care might potentially be improved by further optimizing the treatment of distal and locally advanced rectal cancer.

**What does this paper add to the literature?**

Evaluation of rectal cancer care in two European countries showed that patients with locally advanced disease were treated in almost all hospitals despite the fact that both countries have referral centres. Care might be improved by further optimizing the treatment of locally advanced and distal rectal cancer, requiring a degree of specialization for certain hospitals.

## Introduction

Evaluation of rectal cancer care at regional, national and international level can provide relevant information regarding current quality, hospital variability, adherence to guidelines and potential areas for improvement 1. In 1995 Sweden was one of the first countries to start a rectal cancer registry 2. Several improvement projects have been launched since then, and these were evaluated using auditing 3. Furthermore, prospective trials were conducted (e.g. Stockholm I–III) that had an impact on the provision of care and guideline development 4. The Netherlands is another northern European country with a similar cultural background and similar developments in rectal cancer care, and there is a long tradition of cooperation between the two countries (e.g. the TME and RAPIDO trials) 5, 6. Colorectal auditing in the Netherlands started relatively late, in 2009, but rapidly evolved to become an important source of quality population‐based information 7.

International comparisons have shown significant differences in the outcomes of colorectal cancer care between countries 8, 9, 10, 11. This would suggest a potential for improvement at a country‐specific level. Sweden is a country with a surface area that is 10.8 times larger than that of the Netherlands but has almost half the number of inhabitants (0.57 times). Recently, there has been a tendency towards reducing the number of hospitals treating rectal cancer. This will have a bigger impact in Sweden than in the Netherlands from a patient perspective due to the travel distance required to access care. Centralization of only complex subpopulations might be an alternative, thereby minimizing the effects on patient logistics. But first we have to analyse in more detail the current management of rectal cancer care in both countries and define potential areas for improvement.

Therefore, the purpose of this international population‐based study was to analyse the predictive factors for the circumferential resection margin (CRM) in the period 2011–2015 after resection of tumour node metastasis (TNM) Stage I–III rectal cancer, using data from the Swedish and Dutch national registries, in which cT1–3 and cT4 stage rectal cancers were separated.

## Method

### Data source

Both northern European national audits, the Swedish ColoRectal Cancer Registry (SCRCR) and the Dutch ColoRectal Audit (DCRA), are disease‐specific, collecting information on patient, tumour, treatment and short‐term outcome characteristics of all patients undergoing surgical resection for primary rectal cancer in academic or peripheral hospitals 2, 12. Currently, all centres in which rectal cancer surgery is performed, in Sweden and the Netherlands, participate in both audits. In contrast to Sweden, a minimum required hospital volume for rectal resections is set at 20 per year in the Netherlands 13.

### Study design

This study compared anonymized data on rectal cancer surgery from the two national population‐based databases. Data were extracted from both registries and merged for analysis based on mandatory key variables needed to calculate performance indicators. This study was conducted in accordance with the STROBE guidelines 14. No ethical approval or informed consent was required under Dutch law as decided by the Medical Ethical Committee, Amsterdam UMC, University of Amsterdam, The Netherlands. For the Swedish part, the study was approved by the Regional Ethics Committee of the Karolinska Institutet in Stockholm, Sweden.

### Patient selection

All patients who underwent resection for primary rectal cancer between 1 January 2011 and 31 December 2015 and were registered in the SCRCR or DCRA were potentially eligible for the study. Minimum data requirements were information on tumour location, height and stage, as well as date of surgery and 30‐day/in‐hospital mortality (*n* = 22 431). Exclusion criteria were synchronous primary colorectal tumours, Stage IV disease and trans‐anal local excision (*n* = 3898). This resulted in 18 533 patients being eligible for analysis.

### Data extraction and definitions

The following data were extracted from the SCRCR and the DCRA databases: patient characteristics [age, gender, American Society of Anesthesiologists (ASA) classification, body mass index (BMI)], tumour characteristics (clinical tumour stage and distance from the anal verge) and diagnostic work‐up [preoperative imaging and multidisciplinary team (MDT) meeting]. There were no specific instructions on measurement of the distance from the anal verge in the DCRA, but this was usually based on either flexible or rigid endoscopy; in the SCRCR it was based on rigid rectoscopy. Preoperative radiotherapy consisted of short‐course radiotherapy (5 × 5 Gy) or chemoradiotherapy (25 × 2 Gy or 28 × 1.8 Gy) with fluoropyrimidine‐based concurrent chemotherapy. Surgical characteristics included: type of procedure, construction of a stoma, setting, approach and multivisceral resection. Pathological outcomes included (y)pTN stage, CRM+ (margin ≤ 1 mm) and number of retrieved (positive) lymph nodes, and stage was defined according to the fifth edition of the TNM classification. The CRM was measured by pathologists using a standardized pathological assessment as introduced during the TME trial in both countries 15. For hospital volume, cut‐off points were used as described in the SCRCR National Quality Report 2016: very low volume (< 12 resections per year), low volume (12–25 resections per year), medium volume (26–50 resections per year) and high volume (> 50 resections per year) 16. Additional analyses were performed using previously published cut‐offs in DCRA analyses (< 20, 20–40, > 40) 17.

### Statistical analysis

Locally advanced rectal cancer was considered to be a distinct clinical entity. For this reason, analyses were performed separately for cT1–3 and cT4 rectal cancer. Although cT3 with a threatened mesorectal fascia also belongs to locally advanced disease, it was not possible to separately identify those patients from the datasets. Categorical or dichotomous outcomes were expressed as absolute numbers with percentages and compared between groups using the Pearson chi‐square test. For continuous variables, the Mann–Whitney *U*‐test was used by their distribution, otherwise the *t*‐test for independent samples was used. Funnel plots with 95% and 99% confidence intervals (CIs) were used to demonstrate the influence of hospital volume for adjusted CRM+ in cT1–3 and cT4 Stage subpopulations. Furthermore, the association between overall hospital volume and other potential predictors of CRM+ was determined by uni‐ and multivariable analysis using binary logistic regression models with the Hosmer–Lemeshow goodness‐of‐fit statistic to check for adequacy of the model. Multivariable models were made for each country separately, because harmonization of data acquisition could not be performed and measurement bias could have been introduced by differences among the countries regarding clinical assessment (e.g. distance to the anal verge, cT4 stage). To assess any influence of time factor, the year of resection (2011–2012, 2013–2015) was added as potential predictor. Factors showing an association in the univariable analysis were included in the multivariable models using a *P*‐value < 0.1. Results were reported in odds ratios (ORs) and 95% CI. Statistical significance was defined as a *P*‐value < 0.05. Statistical analyses were performed in spss 24.0 Statistics for Windows (IBM Corp, Armonk, New York, USA).

## Results

**Figure 1:**
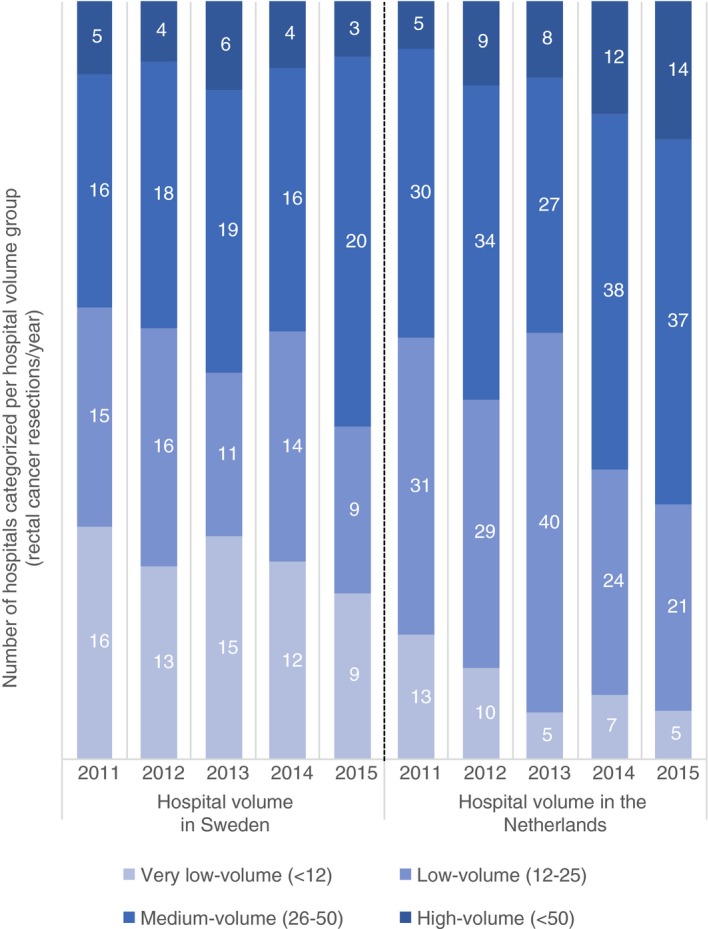
Number of hospitals performing rectal cancer surgery in Sweden and the Netherlands, categorized into four hospital volume groups during the years 2011–2015.

An overview of the two countries concerning the incidence rectal cancer and the data captured in the respective national registries (SCRCR and DCRA) is provided in Table S1. In this study, a total of 18 533 Stage I–III rectal cancer patients between 2011 and 2015 were included for analysis, 6444 from Sweden and 12 089 from the Netherlands. The number of hospitals treating patients with rectal cancer decreased from 52 to 42 in Sweden, and from 82 to 79 in the Netherlands, with a shift to higher volumes in both countries in 2015 (Fig. 1). The number of Stage I–III rectal cancer patients treated in very low‐volume centres during the study period was 314 (4.9%) in Sweden and 201 (1.7%) in the Netherlands; in low‐volume centres the corresponding numbers were 965 (15%) and 2643 (21.9%), in medium‐volume centres 3823 (59.3%) and 6145 (50.8%) and in high‐volume centres 1342 (20.8%) and 3100 (25.6%) patients, respectively.

The patient‐, tumour‐ and treatment characteristics are displayed by country in Table 1, and subdivided for the four hospital volume groups in Table S2. Compared with the other hospital volume categories in Sweden, high‐volume hospitals treated significantly more patients below the age of 75 years (72.2%, *P* < 0.001), more ASA class III patients (27.0%, *P* = 0.021), more with BMI ≤ 30 (86.2%, *P* = 0.042), and fewer with rectal tumours ≤ 5 cm (30.5%, *P* = 0.022) and cT4 stage (22.7%, *P* < 0.001). The same differences for age and cT4 stage were observed for the high‐volume hospitals compared with the lower volume hospitals in the Netherlands. Within high‐volume hospitals, the Netherlands had a significantly lower proportion of cT4 tumours compared with Sweden (9.5% *vs* 22.7%, *P* < 0.001), although the absolute numbers of patients were similar (302 *vs* 294). Overall, a significantly higher proportion of patients in Sweden underwent surgery without preoperative radiotherapy compared with the Netherlands (34.9% *vs* 26.4%, *P* < 0.001). Laparoscopic surgery was less often performed in Sweden than the Netherlands (20.9% *vs* 69.7%, *P* < 0.001), with a higher conversion rate (19.2% *vs* 10.7%, *P* < 0.001). Rectal surgery consisted more often of abdominoperineal excision (APR) procedures in Sweden (36.6% *vs* 30.5%, *P* < 0.001), whereas a higher proportion of Hartmann’s procedures were performed in the Netherlands (16.0% *vs* 10.6%, *P* < 0.001).

**Table 1:** Patient, tumour and treatment characteristics and pathological outcomes for rectal cancer in Sweden and the Netherlands, 2011–2015.

Patient	Sweden	The Netherlands	*P*‐value
	No. of patients	No. of patients	
	(*n* = 6444)	(*n* = 12 089)	
Gender^1^			
Male	3881/6444 (60.2%)	7623/12 082 (63.1%)	**< 0.001**
Female	2563/6444 (39.8%)	4459/12 082 (36.9%)	
Age^2^ (years)			
< 75	4416/6443 (68.5%)	8730/12 084 (72.2%)	**< 0.001**
≥ 75	2027/6443 (31.5%)	3354/12 084 (27.8%)	
ASA^3^			
I–II	4857/6374 (76.2%)	10 116/12 085 (83.7%)	**< 0.001**
III+	1517/6374 (23.8%)	1969/12 085 (16.3%)	
BMI^4^ (kg/m^2^)			
< 30	5203/6223 (83.6%)	9836/11 769 (83.6%)	**0.954**
≥ 30	1020/6223 (16.4%)	1933/11 769 (16.4%)	
Tumour characteristics			
Distance from anal verge^5^ (cm)			
≤ 5	1872/6360 (29.4%)	4435/11 627 (38.1%)	**< 0.001**
6–10	2587/6360 (40.7%)	4527/11 627 (38.9%)	
> 10	1901/6360 (29.9%)	2665/11 627 (22.9%)	
cT stage^6^			
cT1–2	1629/6358 (25.6%)	3481/12 081 (28.8%)	**< 0.001**
cT3	3402/6358 (53.5%)	7061/12 081 (58.4%)	
cT4	1057/6358 (16.6%)	974/12 081 (8.1%)	
cTX/unknown	270/6358 (4.2%)	565/12 081 (4.7%)	
cN stage^7^			
cN0	2642/6439 (41.2%)	5128/12 039 (42.6%)	**< 0.001**
cN1–2	3342/6439 (52.2%)	6273/12 039 (52.1%)	
cNX/unknown	424/6439 (6.6%)	638/12 039 (5.3%)	
Work‐up			
Preoperative pelvic imaging^8^			
Yes*	6358/6444 (98.7%)	11 803/12 032 (98.1%)	**0.007**
MRI	NA	11 272/12 032 (93.7%)	
CT	NA	481/12 032 (4.0%)	
Preoperative MDT meeting^9^			
Yes	6297/6443 (97.3%)	11 898/12 087 (98.4%)	**< 0.001**
Preoperative radiotherapy			
No	2261/6475 (34.9%)	3189/12 089 (26.4%)	**< 0.001**
SCRT	3008/6475 (46.5%)	4518/12 089 (37.4%)	
CRT	1206/6475 (18.6%)	4382/12 089 (36.2%)	
Preoperative chemotherapy			
Yes	273/6444 (4.2%)	135/12 089 (1.1%)	**< 0.001**
Surgery			
Year of operation			
2011–2012	2581/6444 (40.1%)	4416/12 089 (36.5%)	**0.001**
2013–2015	3863/6444 (59.9%)	7673/12 089 (63.5%)	
Annual hospital volume†			
< 12 resections	314/6444 (4.9%)	201/12 089 (1.7%)	**< 0.001**
12–25 resections	965/6444 (15.0%)	2643/12 089 (21.9%)	
26–50 resections	3823/6444 (59.3%)	6145/12 089 (50.8%)	
> 50 resections	1342/6444 (20.8%)	3100/12 089 (25.6%)	
Procedure			
(L)AR	3253/6444 (50.5%)	6340/12 089 (52.4%)	**< 0.001**
Low Hartmann	686/6444 (10.6%)	1937/12 089 (16.0%)	
APR	2359/6444 (36.6%)	3693/12 089 (30.5%)	
Other‡	146/6444 (2.3%)	119/12 089 (1.0%)	
Setting			
Elective	6368/6442 (98.8%)	11 943/12 080 (98.9%)	0.917
Emergency	74/6442 (1.1%)	137/12 080 (1.1%)	
Approach			
Open	5050/6384 (79.1%)	3621/11 958 (30.3%)	**< 0.001**
Laparoscopic	1334/6384 (20.9%)	8337/11 958 (69.7%)	
Laparoscopic conversion	256/1334 (19.2%)	891/8337 (10.7%)	
Intra‐operative bowel perforation^10^			
Yes	302/6309 (4.8%)	107/11 527 (0.9%)	**< 0.001**
Multivisceral resection^11^			
Yes	917/6437 (14.2%)	766/11 761 (6.5%)	**< 0.001**
Pathology			
(y)pT stage^12^			
pT0	221/6360 (3.5%)	923/12 083 (7.7%)	**< 0.001**
pT1	496/6360 (7.8%)	1185/12 083 (9.7%)	
pT2	1840/6360 (28.9%)	3941/12 083 (32.4%)	
pT3	3324/6360 (52.3%)	5426/12 083 (45.0%)	
pT4	433/6360 (6.8%)	447/12 083 (3.8%)	
pTX	46/6360 (0.7%)	127/12 083 (1.1%)	
Unknown	0/6360 (0%)	34/12 083 (0.3%)	
(y)pN stage^13^			
pN0	3906/6362 (61.4%)	8087/12 086 (66.9%)	**< 0.001**
pN1	1595/6362 (25.1%)	2666/12 086 (22.1%)	
pN2	779/6362 (12.2%)	1220/12 086 (10.1%)	
pNX	82/6362 (1.3%)	100/12 086 (0.8%)	
Unknown	0/6362 (0%)	13/12 086 (0.1%)	
CRM overall^14^			
Positive (≤ 1 mm)§	456/5887 (7.8%)	611/11 950 (5.4%)	**< 0.001**
Negative (> 1 mm)	5407/5887 (92.2%)	10 747/11 950 (94.6%)	
CRM cT1–3^15^			
Positive (≤ 1 mm)	280/4651 (6.0%)	465/9963 (4.7%)	**0.001**
Negative (> 1mm)	4371/4651 (94.0%)	9498/9963 (95.3%)	
CRM cT4^16^			
Positive (≤ 1 mm)	142/926 (15.3%)	115/905 (12.7%)	0.106
Negative (> 1mm)	784/926 (84.7%)	790/905 (87.3%)	
Number of lymph nodes retrieved^17^			
> 10	5457/6475 (84.7%)	8543/12 062 (70.8%)	**< 0.001**
Positive lymph nodes^18^			
Yes	2127/6444 (33.0%)	3780/12 062 (31.3%)	**< 0.001**

(L)AR, (low) anterior resection; APR, abdominoperineal excision; ASA, American Society of Anesthesiologists classification; BMI, body mass index; CRM, circumferential resection margin; CRT, chemoradiotherapy; cTNM, clinical tumour–nodal–metastasis; low Hartmann, total mesorectal excision with end‐colostomy; MDT, multidisciplinary team; NA, not applicable; pTNM, pathological tumour–nodal–metastasis; SCRT, short‐course radiotherapy.

Notes: ^1^missing in 7 cases; ^2^missing in 6 cases; ^3^missing in 74 cases; ^4^missing in 541 cases; ^5^missing in 580 cases; ^6^missing in 94 cases; ^7^missing in 86 cases; ^8^missing in 57 cases; ^9^missing in 3 cases; ^10^missing in 697 cases; ^11^missing in 335 cases; ^12^missing in 90 cases; ^13^missing in 85 cases; ^14^missing in 1312 cases; ^15^missing in 959 cases; ^16^missing in 200 cases; ^17^missing in 27 cases; ^18^missing in 27 cases.

* Pelvic imaging: including CT and MRI.

^†^ Only stage TNM Stage I–III rectal cancer patients who underwent rectal resection included.

^‡^ ‘Other’ included total colectomy and proctocolectomy.

^§^ Exclusion of complete response (ypT0) and unknown CRM status.

Usage of bold values is to show the significant *P*‐values (*P* < 0.05).

### CRM+

**Figure 2:**
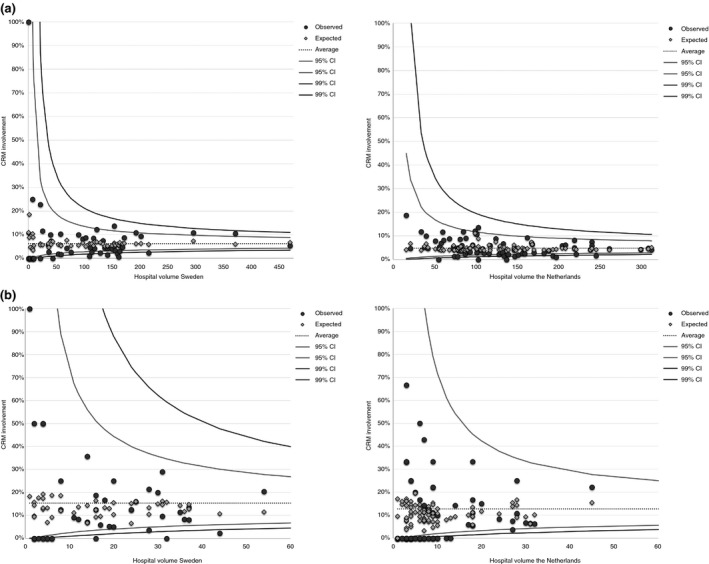
(a) Funnelplot of CRM involvement for case mix‐corrected Swedish/Dutch hospitals performing rectal cancer (cT1–3) surgery (2011–2015). The following factors were included to correct for differences in case mix between patients: sex, BMI, pathological T status, setting, approach and multivisceral resection, preoperative radiotherapy. (b) Funnelplot of CRM involvement for case mix‐corrected Swedish/Dutch hospitals performing rectal cancer (cT4) surgery (2011–2015). The following factors were included to correct for differences in case mix between patients: sex, BMI, pathological T status, setting, approach and multivisceral resection, preoperative radiotherapy.

The overall proportion of CRM+ was 7.8% in Sweden and 5.4% in the Netherlands. CRM+ for cT1–3 tumours was 6.0% and 4.7%, and for cT4 tumours 15.3% and 12.7%, respectively (Table 1). CRM+ relative to the cumulative hospital volume during the study period for each of the Swedish hospitals (Fig. 2a) and Dutch hospitals (Fig. 2b) is depicted in funnel plots. For cT1–3 tumours, CRM+ in medium‐volume hospitals was 5.5% in Sweden and 4.0% in the Netherlands. CRM+ rates in high‐volume hospitals were 7.8% and 5.0%, respectively. For cT4 stage tumours similar CRM+ rates were observed for all the hospital volume groups and both countries.

The results of the uni‐ and multivariable analysis for CRM+ in each of the two countries are demonstrated in Tables 2 and 3. For cT1–3 rectal cancer patients in Sweden, a short distance from the anal verge (≤ 5 cm), cT3 and unknown T stage, APR and multivisceral resection were identified as significant risk factors for CRM+ in multivariable analyses (Table 3a). For the Dutch population with cT1–3 stage rectal cancer, cT3, cN1–2, low Hartmann’s procedure, APR, multivisceral resection, emergency setting and year 2011–2012 were identified as significant risk factors for CRM+, with hospital volumes above 20 being a favourable factor. Common independent risk factors for CRM+ in cT1–3 rectal cancer in both countries were cT3 stage, APR and multivisceral resection. For cT4 rectal cancer, independent risk factors for CRM+ in Sweden were low Hartmann’s procedure and APR. The only independent risk factor for CRM+ in the Netherlands was multivisceral resection. No common risk factors were found in both countries for CRM+ in cT4 stage tumours in multivariable analysis (Table 3b).

**Table 2:** (a) Univariable analysis of predictors of CRM involvement for cT1–3 rectal tumours in Sweden and the Netherlands. (b) Univariable analysis of predictors of CRM involvement for cT4 rectal tumours in the Netherlands and Sweden.

	Sweden			The Netherlands		
	Univariable analysis			Univariable analysis		
	CRM+	OR (95% CI)	*P*‐value	CRM+	OR (95% CI)	*P*‐value
(a)						
Gender						
Male	6.0%	1.12 (0.89–1.42)	0.344	4.7%	0.99 (0.82–1.19)	0.907
Female	6.6%	1.00 (ref)	–	4.7%	1.00 (ref)	–
BMI (kg/m^2^)						
< 30	6.3%	1.00 (ref)	–	4.9%	1.00 (ref)	–
≥ 30	5.8%	0.91 (0.66–1.25)	0.555	4.4%	0.89 (0.69–1.14)	0.350
Distance from the anal verge (cm)						
≤ 5	10.2%	**2.18 (1.62**–**2.94)**	**< 0.001**	6.0%	**1.61 (1.25**–**2.07)**	**< 0.001**
6–10	4.4%	0.88 (0.64–1.21)	0.434	4.0%	1.05 (0.81–1.37)	0.713
> 10	4.9%	1.00 (ref)	–	3.8%	1.00 (ref)	–
cT stage						
cT1–2	3.2%	1.00 (ref)	–	3.3%	1.00 (ref)	–
cT3	7.3%	**2.37 (1.72**–**3.25)**	**< 0.001**	5.3%	**1.63 (1.31**–**2.03)**	**< 0.001**
cTX/unknown	10.2%	**3.39 (2.02**–**5.70)**	**< 0.001**	6.2%	**1.92 (1.27**–**2.92)**	**0.002**
cN stage						
cN0	5.2%	1.00 (ref)	–	3.7%	1.00 (ref)	–
cN1–2	6.7%	**1.33 (1.04**–**1.70)**	**0.025**	5.5%	**1.50 (1.23**–**1.81)**	**< 0.001**
cNX/unknown	9.8%	**1.99 (1.30**–**3.06)**	**0.002**	5.6%	**1.51 (1.01**–**2.27)**	**0.045**
Preoperative MDT meeting						
Yes	6.3%	1.00 (ref)	–	4.7%	1.00 (ref)	–
No	3.4%	0.53 (0.17–1.70)	0.289	8.0%	**1.76 (0.94**–**3.27)**	**0.076**
Preoperative radiotherapy						
No	5.1%	1.00 (ref)	–	4.3%	1.00 (ref)	–
SCRT	6.8%	**1.36 (1.05**–**1.77)**	**0.021**	4.0%	0.92 (0.72–1.17)	0.493
CRT	7.4%	**1.49 (1.03**–**2.16)**	**0.036**	5.9%	**1.40 (1.11**–**1.76)**	**0.004**
Procedure						
(L)AR	4.1%	1.00 (ref)	–	3.2%	1.00 (ref)	–
Low Hartmann	6.2%	**1.54 (1.03**–**2.30)**	**0.035**	6.0%	**1.93 (1.50**–**2.49)**	**< 0.001**
APR	9.6%	**2.45 (1.91**–**3.15)**	**< 0.001**	6.8%	**2.23 (1.81**–**2.74)**	**< 0.001**
Other*	3.8%	0.93 (0.12–6.90)	0.941	14.6%	**5.23 (2.79**–**9.82)**	**< 0.001**
Multivisceral resection						
No	5.8%	1.00 (ref)	–	4.4%	1.00 (ref)	–
Yes	10.5%	**1.92 (1.39**–**2.66)**	**< 0.001**	14.7%	**3.76 (2.75**–**5.14)**	**< 0.001**
Setting						
Elective	6.2%	1.00 (ref)	–	4.7%	1.00 (ref)	–
Emergency	6.3%	1.01 (0.13–7.64)	0.995	13.0%	**3.06 (1.70**–**5.52)**	**< 0.001**
Approach						
Open	6.2%	1.00 (ref)	–	6.4%	1.00 (ref)	–
Laparoscopic	6.2%	1.00 (0.74–1.36)	0.988	4.0%	**0.61 (0.50**–**0.74)**	**< 0.001**
Laparoscopic conversion	6.6%	1.08 (0.62–1.89)	0.788	4.6%	**0.70 (0.49**–**1.02)**	**0.063**
Year of procedure						
2011–2012	7.1%	**1.26 (1.00**–**1.60)**	**0.050**	6.3%	**1.66 (1.38**–**1.99)**	**< 0.001**
2013–2015	5.7%	1.00 (ref)	–	3.9%	1.00 (ref)	–
Hospital volume (3 groups))						
Low (< 20)	5.7%	1.00 (ref)	–	7.0%	1.00 (ref)	–
Medium (20–40)	5.6%	0.97 (0.65–1.45)	0.884	4.5%	**0.62 (0.47**–**0.82)**	**0.001**
High (> 40)	7.6%	1.34 (0.89–2.03)	0.167	4.6%	**0.64 (0.48**–**0.85)**	**0.002**
Hospital volume (4 groups)						
Very low (< 12)	7.9%	1.00 (ref)	–	8.3%	1.00 (ref)	–
Low (12–25)	5.6%	0.69 (0.39–1.22)	0.200	5.4%	0.63 (0.36–1.11)	0.108
Medium (26–50)	5.7%	0.71 (0.43–1.17)	0.178	4.1%	**0.47 (0.27**–**0.81)**	**0.007**
High (> 50)	7.9%	1.00 (0.59–1.71)	1.000	5.1%	**0.60 (0.34**–**1.04)**	**0.068**
(b)						
Gender						
Male	16.3%	0.84 (0.59–1.21)	0.350	14.4%	**0.70 (0.47**–**1.05)**	**0.086**
Female	14.1%	1.00 (ref)	–	10.6%	1.00 (ref)	–
BMI (kg/m^2^)						
< 30	15.8%	1.00 (ref)	–	12.9%	1.00 (ref)	–
≥ 30	9.5%	**0.56 (0.29**–**1.07)**	**0.081**	10.6%	0.80 (0.45–1.42)	0.441
Distance from the anal verge (cm)						
≤ 5	17.5%	1.46 (0.92–2.32)	0.109	12.6%	1.07 (0.59–1.91)	0.831
6–10	13.4%	1.07 (0.64–1.79)	0.796	11.4%	0.95 (0.49–1.83)	0.874
> 10	12.7%	1.00 (ref)	–	11.9%	1.00 (ref)	–
cN stage						
cN0	14.1%	1.00 (ref)	–	14.6%	1.00 (ref)	–
cN1–2	15.1%	1.08 (0.69–1.68)	0.748	12.6%	0.84 (0.54–1.32)	0.458
cNX/unknown	22.8%	1.79 (0.86–3.73)	0.118	3.2%	0.19 (0.03–1.48)	0.114
Preoperative MDT meeting						
Yes	15.3%	1.00 (ref)	–	12.6%	1.00 (ref)	–
No	33.3%	2.77 (0.25–30.79)	0.406	25.0%	2.31 (0.46–11.58)	0.309
Preoperative radiotherapy						
No	14.6%	1.00 (ref)	–	21.2%	1.00 (ref)	–
SCRT	17.6%	1.25 (0.66–2.37)	0.489	11.3%	**0.47 (0.23**–**0.98)**	**0.045**
CRT	14.1%	0.96 (0.52–1.78)	0.893	11.7%	**0.49 (0.29**–**0.83)**	**0.008**
Procedure						
(L)AR	10.0%	1.00 (ref)	–	10.4%	1.00 (ref)	–
Low Hartmann	21.0%	**2.38 (1.30**–**4.38)**	**0.005**	16.9%	**1.76 (0.95**–**3.25)**	**0.074**
APR	18.0%	**1.97 (1.27**–**3.04)**	**0.002**	12.2%	1.20 (0.72–2.01)	0.481
Other*	0.0%	1.00	1.00	23.1%	2.59 (0.66–10.13)	0.171
Multivisceral resection						
No	13.8%	1.00 (ref)	–	9.9%	1.00 (ref)	–
Yes	17.4%	1.32 (0.92–1.89)	0.137	16.7%	**1.82 (1.22**–**2.73)**	**0.003**
Setting						
Elective	15.4%	1.00 (ref)	–	12.4%	1.00 (ref)	–
Emergency	11.1%	0.69 (0.09–5.54)	0.725	33.3%	**3.52 (1.04**–**11.88)**	**0.043**
Approach						
Open	15.9%	1.00 (ref)	–	15.2%	1.00 (ref)	–
Laparoscopic	12.6%	0.77 (0.42–1.41)	0.390	8.6%	**0.52 (0.33**–**0.82)**	**0.005**
Laparoscopic conversion	4.8%	0.27 (0.04–1.99)	0.197	13.2%	0.85 (0.37–1.94)	0.695
Year of procedure						
2011–2012	14.3%	0.88 (0.60–1.27)	0.483	14.1%	1.21 (0.81–1.82)	0.347
2013–2015	18.0%	1.00 (ref)	–	12.0%	1.00 (ref)	–
Hospital volume (3 groups)						
Low (< 20)	13.8%	1.00 (ref)	–	7.1%	1.00 (ref)	–
Medium (20–40)	13.1%	0.94 (0.49–1.83)	0.863	12.7%	1.90 (0.66–5.42)	0.233
High (> 40)	18.8%	1.45 (0.74–2.82)	0.276	13.6%	2.04 (0.71–5.90)	0.188
Hospital volume (4 groups)						
Very low (< 12)	21.9%	1.00 (ref)	–	0.0%	1.00 (ref)	–
Low (12–25)	14.8%	0.62 (0.23–1.66)	0.340	11.3%	1.00	0.999
Medium (26–50)	14.1%	0.58 (0.24–1.40)	0.229	13.4%	1.00	0.999
High (> 50)	17.2%	0.75 (0.30–1.81)	0.509	12.6%	1.00	0.999

(L)AR, (low) anterior resection; APR, abdominoperineal excision; BMI, body mass index; CRT, chemoradiotherapy; cTNM, clinical tumour–nodal–metastasis; Low Hartmann, total mesorectal excision with end‐colostomy; MDT, multidisciplinary team; SCRT, short‐course radiotherapy.

* ‘Other’ included total colectomy and proctocolectomy.

Usage of bold values is to show the significant *P*‐values (*P* < 0.05).

**Table 3:** (a) Multivariable analysis of predictors for CRM involvement in cT1–3 rectal cancer in Sweden and the Netherlands. (b) Multivariable analysis of predictors for CRM involvement in cT4 rectal cancer in Sweden and the Netherlands.

	Sweden		The Netherlands	
	Multivariable analysis		Multivariable analysis	
	OR (95% CI)	*P*‐value	OR (95% CI)	*P*‐value
(a)				
Distance from the anal verge (cm)				
≤ 5	**1.59 (1.01–2.51)**	**0.045**	0.90 (0.65**–**1.24)	0.512
6**–**10	0.84 (0.60**–**1.18)	0.305	0.86 (0.65**–**1.14)	0.287
> 10	1.00 (ref)	**–**	1.00 (ref)	**–**
cT stage				
cT1**–**2	1.00 (ref)	**–**	1.00 (ref)	**–**
cT3	**2.63 (1.85–3.74)**	**< 0.001**	**1.35 (1.06–1.73)**	**0.016**
cTX/unknown	**2.87 (1.61–5.10)**	**< 0.001**	1.09 (0.57**–**2.06)	0.801
cN stage				
cN0	1.00 (ref)	**–**	1.00 (ref)	**–**
cN1**–**2	1.14 (0.86**–**1.51)	0.370	**1.28 (1.02–1.61)**	**0.032**
cNX/unknown	1.57 (0.99**–**2.50)	0.055	0.95 (0.54**–**1.67)	0.846
Preoperative MDT meeting				
Yes	1.00 (ref)	**–**	1.00 (ref)	**–**
No	**–** *	**–**	0.81 (0.31**–**2.08)	0.655
Preoperative radiotherapy				
No	1.00 (ref)	**–**	1.00 (ref)	**–**
SCRT	0.84 (0.61**–**1.14)	0.226	1.00 (ref)	**–**
CRT	0.76 (0.49**–**1.18)	0.215	1.13 (0.90**–**1.41)	0.293
Procedure				
(L)AR	1.00 (ref)	**–**	1.00 (ref)	**–**
Low Hartmann	1.40 (0.93**–**2.11)	0.111	**1.66 (1.26–2.19)**	**< 0.001**
APR	**1.69 (1.15–2.47)**	**0.007**	**1.94 (1.45–2.58)**	**< 0.001**
Other†	0.55 (0.07**–**4.24)	0.567	**2.76 (1.14–6.66)**	**0.024**
Multivisceral resection				
No	1.00 (ref)	**–**	1.00 (ref)	**–**
Yes	**1.74 (1.24–2.43)**	**0.001**	**2.62 (1.86–3.70)**	**< 0.001**
Setting				
Elective	1.00 (ref)	**–**	1.00 (ref)	**–**
Emergency	**–**	**–**	**2.84 (1.39–5.82)**	**0.004**
Approach				
Open	1.00 (ref)	**–**	1.00 (ref)	**–**
Laparoscopic	**–** *	**–**	0.87 (0.70**–**1.08)	0.202
Laparoscopic conversion	**–** *	**–**	0.85 (0.57**–**1.26)	0.417
Year of procedure				
2011**–**2012	1.26 (0.99**–**1.62)	0.061	**1.81 (1.47–2.22)**	**< 0.001**
2013**–**2015	1.00 (ref)	**–**	1.00 (ref)	**–**
Hospital volume (3 groups)				
Low (< 20)	1.00 (ref)	**–**	1.00 (ref)	**–**
Medium (20**–**40)	**–** *	**–**	**0.56 (0.42–0.74)**	**< 0.001**
High (> 40)	**–** *	**–**	**0.59 (0.43–0.79)**	**< 0.001**
Hospital volume (4 groups)				
Very low (< 12)	1.00 (ref)	**–**	1.00 (ref)	**–**
Low (12**–**25)	**–** *	**–**	0.82 (0.46**–**1.47)	0.500
Medium (26**–**50)	**–** *	**–**	**0.54 (0.30–0.95)**	**0.032**
High (> 50)	**–** *	**–**	0.72 (0.40**–**1.30)	0.273
(b)				
Gender				
Male	**–** *	**–**	0.65 (0.42**–**1.00)	0.052
Female	1.00 (ref)	**–**	1.00 (ref)	**–**
BMI (kg/m^2^)				
< 30	1.00 (ref)	**–**	1.00 (ref)	**–**
≥ 30	0.58 (0.30**–**1.11)	0.098	**–** *	**–**
Preoperative radiotherapy				
No	1.00 (ref)	**–**	1.00 (ref)	**–**
SCRT	**–** *	**–**	0.89 (0.39**–**2.01)	0.776
CRT	**–** *	**–**	0.75 (0.40**–**1.39)	0.357
Procedure				
(L)AR without DS	1.00 (ref)	**–**	1.00 (ref)	**–**
Low Hartmann	**2.38 (1.28–4.44)**	**0.006**	1.29 (0.67**–**2.51)	0.447
APR	**1.89 (1.21–2.94)**	**0.005**	1.03 (0.60**–**1.75)	0.928
Other†	1.00	0.999	1.80 (0.44**–**7.37)	0.416
Multivisceral resection				
No	1.00 (ref)	**–**	1.00 (ref)	**–**
Yes	**–** *	**–**	**1.77 (1.13–2.79)**	**0.013**
Setting				
Elective	1.00 (ref)	**–**	1.00 (ref)	**–**
Emergency	**–** *	**–**	3.01 (0.66**–**13.75)	0.155
Approach				
Open	1.00 (ref)	**–**	1.00 (ref)	**–**
Laparoscopic	**–** *	**–**	0.70 (0.42**–**1.17)	0.174
Laparoscopic conversion	**–** *	**–**	0.93 (0.39**–**2.20)	0.862

(L)AR, (low) anterior resection; APR, abdominoperineal excision; BMI, body mass index; CRT, chemoradiotherapy; cTNM, clinical tumour–nodal–metastasis; DS, diverting stoma; Low Hartmann, total mesorectal excision with end‐colostomy; MDT, multidisciplinary team; SCRT, short‐course radiotherapy.

* Not tested in multivariable analysis, not significant in univariable analysis.

^†^ ‘Other’ included total colectomy and proctocolectomy.

Usage of bold values is to show the significant *P*‐values (*P* < 0.05).

## Discussion and conclusions

This international population‐based study evaluated rectal cancer care based on CRM in two northern European countries between 2011 and 2015. The Swedish Stage I–III rectal cancer population underwent more multivisceral resections due to more cT4 tumours and more APRs compared with the Netherlands. For cT1–3 tumours, the rate of CRM+ was around 5%, but with an almost threefold increase for cT4 tumours in both countries. The use of laparoscopic surgery differed substantially between the countries, but showed no impact on CRM+. Importantly, CRM+ appeared to be associated with locally advanced disease as well as distal tumours in both countries. This indicates potential areas for improvement, especially given the observation that patients with locally advanced disease were treated in almost all hospitals.

In this study, several significant differences were observed in baseline characteristics between Sweden and the Netherlands that possibly had an effect on CRM+. Performing multivariable analyses for the clinically distinct populations with cT1–3 and cT4 stage rectal cancer within each country revealed, however, that only some of these cross‐country differences were predictive for incomplete resections. High‐risk patients with locally advanced disease were still mostly treated in very low volumes in almost every hospital. This is probably also the reason why no impact of volume could be demonstrated for cT4 rectal cancer in the present study, because this type of care is currently too fragmented among all hospitals. Locally advanced tumours require multimodality treatment with often an extensive surgical procedure to achieve a radical resection 18, 19, 20. Despite preoperative radiotherapy and multivisceral resection, substantial higher CRM+ rates were observed in the cT4 compared with the cT1–3 populations, with large hospital variability. In addition, the large T3 tumours that required multivisceral resection also appeared to be associated with significantly higher risk of CRM+. Interestingly, more awareness of cT4 rectal tumours in Sweden related to a relatively higher incidence in combination with the higher proportion of multivisceral resections did not result in lower CRM+ rates compared with the Netherlands (even nonsignificantly higher). This observation is difficult to explain, but probably emphasizes the difficulty in optimizing treatment of this high‐risk group if there a critical volume is still not reached after which additional expertise is gained and results will eventually improve.

Despite the fact that both countries have national referral centres for locally advanced disease, many such patients were still treated at regional hospitals. Decisions for referral are often based on the response to preoperative treatment. Dumont *et al*. showed that a residual tumour greater than 3 cm and tumour fixity are predictive of CRM+ 21. However, one might argue that every locally advanced tumour should probably be referred from the start of treatment to a specialized centre. This is also supported by recent analysis from the Netherlands Comprehensive Cancer Network, showing improved survival in centres treating cT4 stage rectal cancer in higher volumes (more than 10 cases a year) 22.

The same seems applicable for distal rectal cancer considering distance from the anal verge, APR and low Hartmann’s as risk factors for CRM+ in the present analysis. The introduction of MRI together with optimized surgical techniques have improved the outcome of distal rectal cancer that is mostly only amenable to APR 23, 24, 25. The present data indicate that this is probably not generalizable to the population level, and that there is still room for improvement in patients undergoing APR. Furthermore, sphincter‐preserving surgery for distal rectal cancer might be especially challenging. New minimally invasive approaches have been introduced that aim to improve the outcome of sphincter‐saving resections of distal rectal cancer 26, 27. These complex procedures still have to prove their additional value. Centralization might aid the implementation of new surgical techniques for distal rectal cancer with long learning curves. Assigning a certain number of hospitals throughout the country as referral centres for locally advanced and distal rectal cancer would increase the per centre volume of such patients that require complex treatment. This does not necessarily mean a high‐volume centre for every Stage I–III rectal cancer, but rather a certain degree of specialization.

Low‐volume hospitals (defined as one carrying out 20 rectal resections per year) were identified as a significant predictor for CRM+ in the Dutch cT1–3 population. Using the Swedish volume definitions, medium‐volume hospitals (defined as performing 26–50 rectal resections a year) did significantly better than the other volume groups. It is difficult to set out the appropriate cut‐off point for hospital volume groups. In the international literature, a wide range of categories are used for rectal cancer volume at hospital level. Van Gijn *et al*. calculated median cut‐off points from published rectal cancer hospital volume studies, leading to a definition of a high‐volume hospital a performing more than 24 (17–35) rectal cancer resections per year and low‐volume hospitals performing 9 (6–14) 28. This hampers interpretation of the available literature on this topic. To make it even more complicated, others have looked at surgeon volumes or other infrastructural characteristics such as the existence of structured MDTs or demonstration of appropriate expertise 29.

A basic number of patients is needed to optimize care pathways and improve specific aspects of care, such as work‐up, neoadjuvant therapy, surgical technique and postoperative care of distal and locally advanced rectal cancer. This also facilitates the conduct of clinical trials and translational research. From a patient perspective, centralization for specific subgroups of rectal cancer has implications for access to care, which especially affect the patients in Sweden referred to other hospitals many miles away. Long travel distances may lead to difficulties in seeking care for postoperative complications, but studies have shown that the advantages of treatment at specialized centres outweigh the disadvantages related to long travel distances 30.

The strength of this study is the large numbers of patients and external validity, common to population‐based studies in general. But there are several limitations to be mentioned. These include differences in management of rectal cancer and referral patterns, but details on the care pathways and protocols are not available in the audits, neither are details on the MDTs providing this care. Also, variation in the start of the Bowel Cancer Screening Programme in Sweden (2008), which is only regionally used in the Stockholm region, and the Netherlands (2014) could have led to differences in patient populations, although the data showed higher tumour stages in Sweden 31, 32. Furthermore, data availability, data quality and data completeness in both registries should always be considered when interpreting analyses of registry data in general. Key variables such as tumour location, CRM status and mortality are mandatory in both registries, and completeness of these variables is high, as shown in Table 1. The quality of data in the DCRA has been checked by comparison with the Netherlands Cancer Registry and external validations have been performed using original patient files 12. Data quality and completeness of the SCRC were also evaluated, showing high validity 33. Therefore, we feel that these factors might have had only limited influence on the data. Finally, one should realize that overall rectal cancer volumes are slightly higher because local excisions, multiple synchronous cancers and metastatic disease (Stage IV) were excluded.

This international population‐based study, based on data from the SCRCR and the DCRA, demonstrated that substantial differences exist between Sweden and the Netherlands with regard to rectal cancer care. By analysing CRM involvement with correction for confounders, the data suggest that further optimization of rectal cancer care can potentially be accomplished by focusing on patients with distally located and locally advanced disease.

## Conflicts of interest

RD, DS, MPMdNtB, JWTD, MWJMW, NAWvG, WAB, PJT, AM and MW have no conflicts of interest or financial ties to disclose.

## Availability of data and material

The data that support the findings of this study are available from the Dutch Institute for Clinical Auditing (DICA) but are not publicly available. Data availability is possible upon reasonable request to the authors and with permission of the DICA, the DCRA Board and the SCRCR.

## Preregistration of study or analysis plan disclosure

It is an population based study with audit data of the Dutch ColoRectal Audit. The preregistration / or analysis plan of this study can be find online here: http://dica.nl/dcra/onderzoek

## Supporting information


**Table S1** **.** Comparison of Sweden and the Netherlands with regard to rectal cancer incidence and data captured in the respective national registries.
**Table S2** **.** Patient, tumour and treatment characteristics and pathological outcomes for rectal cancer subdivided by hospital volume groups in Sweden and the Netherlands.
