# Supplementary material for: International evaluation of circumferential resection margins after rectal cancer resection: insights from the Swedish and Dutch audits
Source: Colorectal Dis. 2019 Nov 27;22(4):416–29. doi: 10.1111/codi.14903 (PMC7187294; doi:10.1111/codi.14903)
Supplement: Supplementary file 1 — Table S1 . Comparison of Sweden and the Netherlands with regard to rectal cancer incidence and data captured in the respective national registries. Table S2 . Patient, tumour and treatment characteristics and pathological outcomes for rectal cancer subdivided by hospital volume groups in Sweden and the Netherlands. [file CODI-22-416-s001.docx]

**APPENDICES**

**Appendix Table A.1.** Comparison of Sweden and the Netherlands with regard to rectal cancer incidence and data captured in the respective national registries.

|  |  | **Sweden** | **The Netherlands** |
| --- | --- | --- | --- |
| **Inhabitants (x10^6) (2015)** |  | 9.80 | 16.94 |
| **Incidence (2015)** | Rectal cancer | 2095 | 4803 |
| **Minimal annual volume** |  | No | 2012: 20 rectal resections/year/hospital independent of underlying disease |
| **Registry** |  |  |  |
|  | Registry name | Swedish ColoRectal Cancer Registry (SCRCR) | Dutch ColoRectal Audit (DCRA) |
|  | Registry type | Clinical audit | Clinical audit |
|  | Registry active since | 1995 | 2009 |
|  | Data collection | Physicians/Regional Cancer Centre (RCC) | Surgeons/Specialized nursing or administrative staff/Netherlands Cancer Registry (NCR) |
|  | Years of diagnosis in dataset | 2011-2015 | 2011-2015 |
|  | Case ascertainment | Nationwide | Nationwide |
|  | Mandatory | Yes | Yes |
|  | Patients included | All patients | Local excision or rectal resection for primary rectal cancer |
| **Data availability** |  |  |  |
| **Patient characteristics** | Age, sex, BMI | +/+/+ | +/+/+ |
|  | Co-morbidity (ASA/Charlson) | +/- | +/+ |
| **Tumour characteristics** | Location | + | + |
|  | Histology/differentiation | + | + |
|  | cTNM-stage | + | + |
| **Diagnostics** | Preoperative imaging  (CT, MRI) | + | ++ |
|  | Preoperative MDT-meeting | + | + |
| **Neoadjuvant therapy** | Preoperative (chemo)radiotherapy | + | + |
|  | Preoperative chemotherapy not as part of radiotherapy schedule | + | + |
| **Surgical treatment** | Procedure | + | + |
|  | Minimal invasive | + | + |
|  | Primary anastomosis/stoma formation | +/+ | +/+ |
|  | Conversion | + | + |
|  | Urgency (emergency, elective) | + | + |
| **Pathology** | (y)pTNM-stage | + | + |
|  | Response to treatment | - | + |
|  | Number of (positive) lymph nodes | + | + |
|  | Circumferential resection margin (CRM) | + | + |
|  | Distal resection margin (DRM) | - | Until 2015 |
| **Post-operative course** | Post-operative complications | + | + |
|  | Severity of complication | + | + |
|  | Re-intervention (<30 days) | + | + |
|  | Readmission (<30-days) | + | + |
| **30-day and in-hospital mortality** |  | + | + |
| **90-day mortality** |  | + | - |

BMI = Body Mass Index, ASA = American Society of Anaesthesiologists-Classification, TNM= Tumour-Nodal-Metastasis, CT= Computer Tomography, MRI= Magnetic Resonance Imaging, MDT= Multidisciplinary Team. Data available from the Netherlands Cancer Registry and the Central Cancer Registry.

**Appendix Table A.2.** Patient, tumour and treatment characteristics and pathological outcomes for rectal cancer subdivided in hospital volume groups in Sweden and the Netherlands

|  |  |  | **Sweden** | |  |  |  | **the Netherlands** | |  |  |  |
| --- | --- | --- | --- | --- | --- | --- | --- | --- | --- | --- | --- | --- |
|  |  | **Very low-volume** | **Low-volume** | **Medium-volume** | **High-volume** | **P-value** | **Very low-volume** | **Low-volume** | **Medium-volume** | **High-volume** | **P-value** | **P-value** |
| **Patient*** |  | Number of patients | Number of patients | Number of patients | Number of patients | Hospital volume Sweden | Number of patients | Number of patients | Number of patients | Number of patients | Hospital volume the Netherlands | Hospital volume Sweden vs. the Netherlands |
|  |  | (n=314) | (n=965) | (n=3823) | (n=1342) |  | (n=201) | (n=2643) | (n=6145) | (n=3100) |  |  |
| **Gender** | Male | 179 (57.0) | 567 (58.8) | 2327 (60.9) | 808 (60.2) | 0.409 | 114 (56.7) | 1639 (62.0) | 3879 (63.2) | 1991 (64.2) | 0.087 | **0.028** |
|  | Female | 135 (43.0) | 398 (41.2) | 1496 (39.1) | 534 (39.8) |  | 87 (43.3) | 1003 (38.0) | 2261 (36.8) | 1108 (35.8) |  |  |
| **Age** | <75 | 192 (61.1) | 623 (64.6) | 2633 (68.9) | 968 (72.2) | **<0.001** | 130 (65.0) | 1878 (71.1) | 4439 (72.2) | 2283 (73.7) | **0.018** | **<0.001** |
|  | ≥75 | 122 (38.9) | 342 (35.4) | 1190 (31.1) | 373 (27.8) |  | 70 (35.0) | 763 (28.9) | 1705 (27.8) | 816 (26.3) |  |  |
| **ASA** | I - II | 229 (76.8) | 726 (76.2) | 2929 (77.3) | 973 (73.0) | **0.021** | 168 (83.6) | 2208 (83.6) | 5162 (84.0) | 2578 (83.2) | 0.772 | 0.170 |
|  | III+ | 69 (23.2) | 227 (23.8) | 862 (22.7) | 359 (27.0) |  | 33 (16.4) | 432 (16.4) | 982 (16.0) | 522 (16.8) |  |  |
| **BMI** | <30 | 247 (83.2) | 744 (83.3) | 3077 (82.8) | 1135 (86.2) | **0.042** | 161 (80.9) | 2185 (84.1) | 4929 (83.3) | 2566 (83.9) | 0.539 | 0.126 |
|  | ≥30 | 50 (16.8) | 149 (16.7) | 639 (17.2) | 182 (13.8) |  | 38 (19.1) | 413 (15.9) | 989 (16.7) | 493 (16.1) |  |  |
| **Tumour** |  |  |  |  |  |  |  |  |  |  |  |  |
| **Distance from anal verge** | ≤5 cm | 65 (21.3) | 293 (30.7) | 1112 (29.4) | 402 (30.5) | **0.024** | 71 (36.6) | 937 (36.6) | 2317 (38.7) | 1110 (38.5) | 0.119 | **0.001** |
|  | 6-10 cm | 126 (41.3) | 382 (40.1) | 1555 (41.1) | 524 (39.7) |  | 76 (39.2) | 994 (38.8) | 2366 (39.5) | 1091 (37.9) |  |  |
|  | >10 cm | 114 (37.4) | 278 (29.2) | 1115 (29.5) | 394 (29.8) |  | 47 (24.2) | 628 (24.5) | 1310 (21.9) | 680 (23.6) |  |  |
| **cT stage** | cT1-2 | 80 (26.2) | 261 (27.3) | 936 (24.8) | 352 (26.64) | **<0.001** | 71 (35.7) | 817 (30.9) | 1602 (26.1) | 991 (32.0) | **<0.001** | **<0.001** |
|  | cT3 | 151 (49.5) | 491 (51.4) | 2153 (57.2) | 607 (45.6) |  | 106 (53.3) | 1575 (59.6) | 3666 (59.7) | 1714 (55.4) |  |  |
|  | cT4 | 41 (13.4) | 138 (14.5) | 576 (15.3) | 302 (22.7) |  | 9 (4.5) | 149 (5.6) | 522 (8.5) | 294 (9.5) |  |  |
|  | cTX / unknown | 33 (10.8) | 65 (6.8) | 102 (2.7) | 70 (5.3) |  | 13 (6.5) | 102 (3.9) | 354 (5.8) | 96 (3.1) |  |  |
| **cN stage** | cN0 | 160 (51.4) | 402 (41.9) | 1524 (40.1) | 556 (41.7) | **<0.001** | 83 (41.7) | 1289 (48.8) | 2485 (40.7) | 1271 (41.2) | **<0.001** | **<0.001** |
|  | cN1-2 | 133 (42.8) | 431 (44.9) | 2151 (56.6) | 627 (47.0) |  | 104 (52.3) | 1237 (46.8) | 3235 (52.9) | 1697 (55.0) |  |  |
|  | cNX ‎/ unknown | 18 (5.8) | 127 (13.2) | 128 (3.4) | 151 (11.3) |  | 12 (6.0) | 117 (4.4) | 390 (6.4) | 119 (3.9) |  |  |
| **Work-up** |  |  |  |  |  |  |  |  |  |  |  |  |
| **Pre-operative pelvic imaging** | Yes** | 304 (96.8) | 955 (99.0) | 3768 (98.6) | 1331 (99.2) | **0.008** | 198 (99.0) | 2567 (97.5) | 6021 (98.4) | 3017 (97.9) | **0.020** | 0.085 |
|  | MRI | NA | NA | NA | NA |  | 187 (93.5) | 2426 (92.1) | 5724 (96.5) | 2935 (95.3) |  |  |
|  | CT | NA | NA | NA | NA |  | 9 (1.0) | 132 (5.0) | 275 (4.5) | 65 (2.1) |  |  |
| **Pre-operative MDT-meeting** | Yes | 278 (88.5) | 902 (93.5) | 3766 (98.5) | 1325 (98.8) | **<0.001** | 196 (97.5) | 2587 (98.0) | 6064 (98.7) | 3051 (98.4) | **0.030** | **<0.001** |
| **Neoadjuvant radiotherapy** | No | 160 (51.0) | 352 (36.5) | 1221 (31.9) | 503 (37.5) | **<0.001** | 43 (21.4) | 750 (28.4) | 1553 (25.3) | 843 (27.2) | **<0.001** | **<0.001** |
|  | SCRT | 125 (39.8) | 468 (48.5) | 1898 (49.6) | 512 (38.2) |  | 87 (43.3) | 1043 (39.5) | 2156 (35.1) | 1232 (39.7) |  |  |
|  | CRT | 29 (9.2) | 145 (15.0) | 704 (18.4) | 327 (24.4) |  | 71 (35.3) | 850 (32.2) | 2436 (39.6) | 1025 (33.1) |  |  |
| **Surgery** |  |  |  |  |  |  |  |  |  |  |  |  |
| **Year of operation** | 2011-2012 | 192 (61.1) | 406 (42.1) | 1451 (38.0) | 532 (39.6) | **<0.001** | 110 (54.7) | 960 (36.3) | 2294 (37.3) | 1052 (33.9) | **<0.001** | **<0.001** |
|  | 2013-2015 | 122 (38.9) | 559 (57.9) | 2372 (62.0) | 810 (60.4) |  | 91 (45.3) | 1683 (63.7) | 3851 (62.7) | 2048 (66.1) |  |  |
| **Procedure** | (L)AR | 176 (56.1) | 495 (51.3) | 1992 (51.3) | 590 (44.0) | **<0.001** | 101 (50.2) | 1508 (57.1) | 3190 (51.9) | 1541 (49.7) | **<0.001** | **<0.001** |
|  | Low Hartmann | 47 (15.0) | 111 (11.5) | 376 (9.8) | 152 (11.3) |  | 31 (15.4) | 407 (15.4) | 1031 (16.8) | 468 (15.1) |  |  |
|  | APR | 74 (23.6) | 342 (35.4) | 1376 (36.0) | 567 (42.3) |  | 64 (31.8) | 703 (26.6) | 1864 (30.3) | 1062 (34.3) |  |  |
|  | Other*** | 17 (5.4) | 17 (1.8) | 79 (2.1) | 33 (2.5) |  | 5 (2.5) | 25 (0.9) | 60 (1.0) | 29 (0.9) |  |  |
| **Setting** | Elective | 303 (96.5) | 956 (99.1) | 3780 (98.9) | 1329 (99.0) | **0.001** | 190 (95.0) | 2610 (98.9) | 6087 (99.1) | 3056 (98.6) | **<0.001** | **<0.001** |
|  | Emergency | 11 (3.5) | 9 (0.9) | 41 (1.1) | 13 (1.0) |  | 10 (5.0) | 29 (1.1) | 55 (0.9) | 43 (1.4) |  |  |
| **Approach** | Open | 283 (91.3) | 791 (82.9) | 2895 (76.3) | 1081 (81.6) | **<0.001** | 67 (33.7) | 765 (29.4) | 2035 (33.4) | 754 (24.6) | **<0.001** | **<0.001** |
|  | Laparoscopic | 25 (8.1) | 147 (15.4) | 712 (18.8) | 194 (14.7) |  | 110 (55.3) | 1591 (61.2) | 3643 (59.8) | 2102 (68.6) |  |  |
|  | Laparoscopic conversion | 2 (8.0) | 16 (10.9) | 189 (26.5) | 49 (25.3) |  | 22 (20.0) | 244 (15.3) | 416 (11.4) | 209 (9.9) |  |  |
| **Intraoperative bowel perforation** | Yes | 13 (4.4) | 52 (5.5) | 153 (4.1) | 84 (6.3) | **0.007** | 3 (1.6) | 19 (0.7) | 43 (0.7) | 42 (1.4) | **0.007** | **0.003** |
| **Multivisceral resection** | Yes | 37 (11.8) | 108 (11.2) | 464 (12.2) | 308 (23.0) | **<0.001** | 12 (6.0) | 104 (3.9) | 459 (7.7) | 191 (6.5) | **<0.001** | **<0.001** |
| **Pathology** |  |  |  |  |  |  |  |  |  |  |  |  |
| **(y)pT stage^12^** | pT0 | 1 (0.3) | 19 (2.0) | 151 (4.0) | 50 (3.8) | **<0.001** | 26 (12.9) | 195 (7.4) | 500 (8.1) | 202 (6.5) | **<0.001** | **<0.001** |
|  | pT1 | 27 (8.8) | 73 (7.7) | 319 (8.5) | 77 (5.8) |  | 24 (11.9) | 262 (9.9) | 616 (10.0) | 283 (9.1) |  |  |
|  | pT2 | 81 (26.5) | 290 (30.5) | 1128 (29.9) | 341 (25.6) |  | 48 (23.9) | 889 (33.7) | 2018 (32.9) | 1078 (31.8) |  |  |
|  | pT3 | 170 (55.6) | 510 (53.6) | 1926 (51.1) | 718 (53.9) |  | 98 (48.8) | 1189 (45.1) | 2675 (43.5) | 1464 (47.2) |  |  |
|  | pT4 | 23 (7.5) | 52 (5.5) | 222 (5.9) | 136 (10.2) |  | 4 (2.0) | 84 (3.2) | 252 (4.1) | 107 (3.5) |  |  |
|  | pTX | 4 (1.3) | 8 (0.8) | 23 (0.6) | 11 (0.8) |  | 0 (0) | 12 (0.5) | 66 (1.1) | 49 (1.6) |  |  |
|  | Unknown | 0 (0) | 0 (0) | 0 (0) | 0 (0) |  | 1 (0.5) | 8 (0.3) | 16 (0.3) | 10 (0.3) |  |  |
| **(y)pN stage^13^** | pN0 | 186 (60.8) | 560 (58.8) | 2387 (63.3) | 773 (58.0) | **<0.001** | 134 (66.7) | 1753 (66.4) | 4137 (67.3) | 2063 (66.5) | **0.029** | **0.002** |
|  | pN1 | 70 (22.9) | 231 (24.2) | 918 (24.4) | 376 (28.2) |  | 54 (26.9) | 567 (21.5) | 1320 (21.5) | 725 (23.4) |  |  |
|  | pN2 | 41 (13.4) | 142 (14.9) | 429 (11.4) | 167 (12.5) |  | 13 (6.5) | 289 (10.9) | 625 (10.2) | 293 (9.5) |  |  |
|  | pNX | 9 (2.9) | 20 (2.1) | 36 (1.0) | 17 (1.3) |  | 0 (0) | 31 (1.2) | 54 (0.9) | 15 (0.5) |  |  |
|  | Unknown | 0 (0) | 0 (0) | 0 (0) | 0 (0) |  | 0 (0) | 2 (0.1) | 7 (0.1) | 4 (0.1) |  |  |
| **CRM overall^14^** | Positive (≤1 mm)**** | 28 (10.6) | 59 (6.9) | 245 (7.0) | 124 (10.1) | **0.001** | 16 (8.4) | 143 (5.8) | 282 (4.9) | 170 (5.8) | **0.043** | **<0.001** |
|  | Negative (>1 mm) | 237 (89.4) | 802 (93.1) | 3260 (93.0) | 1108 (89.9) |  | 174 (91.6) | 2343 (94.2) | 5492 (95.1) | 2738 (94.2) |  |  |
| **CRM cT1-3^15^** | Positive (≤1 mm)**** | 18 (9.0) | 34 (5.0) | 158 (5.5) | 70 (7.8) | **0.012** | 15 (8.7) | 124 (5.5) | 199 (4.0) | 127 (5.0) | **0.002** | **<0.001** |
|  | Negative (>1 mm) | 183 (91.0) | 649 (95.0) | 2715 (94.5) | 824 (92.2) |  | 158 (91.3) | 2146 (94.5) | 4782 (96.0) | 2412 (95.0) |  |  |
| **CRM cT4^16^** | Positive (≤1 mm)**** | 7 (21.9) | 17 (14.8) | 71 (14.1) | 47 (17.2) | 0.491 | 0 (0.0) | 15 (11.3) | 65 (13.4) | 35 (12.6) | 0.658 | 0.789 |
|  | Negative (>1 mm) | 25 (78.1) | 98 (85.2) | 434 (85.9) | 227 (82.8) |  | 8 (100) | 118 (88.7) | 421 (86.6) | 243 (87.4) |  |  |
| **Number of lymph nodes retrieved^17^** | >10 | 253 (80.6) | 793 (82.2) | 3209 (83.9) | 1202 (89.6) | **<0.001** | 123 (61.8) | 1904 (72.2) | 4295 (70.0) | 2221 (71.8) | **0.028** | **<0.001** |
| **Positive lymph nodes^18^** | Yes | 104 (33.1) | 339 (35.1) | 1217 (31.8) | 467 (34.8) | **<0.001** | 66 (33.2) | 840 (31.9) | 1876 (30.6) | 998 (32.3) | 0.061 | **<0.001** |

ASA=American Society of Anaesthesiologists-Classification, BMI=Body Mass Index, cTNM= clinically Tumour-Nodal-Metastasis, MRI= Magnetic Resonance Imaging, CT= Computer Tomogram, NA= Not Applicable, MDT=Multi-Disciplinary Team, (L)AR=(low) anterior resection, Low Hartmann= total mesorectal excision with end-colostomy, APR=abdominoperineal resection, Low Hartmann= total mesorectal excision with end-colostomy. pTNM= pathologically Tumour-Nodal-Metastasis, CRM=Circumferential Resection Margin. 1=missing in 7 cases, 2=missing in 6 cases, 3=missing in 74 cases, 4=missing in 541 cases, 5=missing in 580 cases, 6=missing in 94 cases, 7=missing in 86 cases, 8= missing in 57 cases, 9=missing in 3 cases, 10= missing in 697 cases, 11=missing in 335 cases, 12=missing in 90 cases, 13= missing in 85 cases. 14= missing in 1312 cases, 15= missing in 959 cases, 16= missing in 200 cases, 17= missing in 27 cases, 18=missing in 27 cases.

*Note: only stage TNM stage I-III rectal cancer patients who underwent rectal resection included. **Note: pelvic imaging: including CT and MRI. ***Note: “other” included total colectomy and proctocolectomy. ****Note: exclusion of complete response (ypT0) and unknown CRM status.
